# The relationship between terrain and rural migration (1965–2013) on the north of Turkey (the case of Kastamonu)

**DOI:** 10.1007/s10661-017-5867-9

**Published:** 2017-03-09

**Authors:** Seda Erkan Buğday, Sezgin Özden

**Affiliations:** 1 Faculty of Forestry, Kastamonu University, 37150 Kastamonu, Turkey; 2 Faculty of Forestry, Cankiri Karatekin University, 18100 Çankırı, Turkey

**Keywords:** Forest villages, Migration, Spatial analysis, Turkey, Kastamonu

## Abstract

Migration is one of the most important issues in Turkey today. Notably, the state forest enterprises are affected by the social, economic, and ecological dimensions of migration; these enterprises find it increasingly difficult to find labor to operate effectively in the forestry activities with each passing day. This study examined population movement over the 1965–2013 period in Kastamonu forest villages to assess how topographical factors affected this movement. Kastamonu is the province in which most of Turkey’s forest products are produced, and 99% of the province’s rural population consists of forest villagers. This study investigated population fluctuations of 883 villages within and 137 villages adjacent to these forests and found a negative linear tendency in these populations. The purpose of this study is to identify a relationship between the decreasing population and the terrain of the forest villages (including aspect, slope, elevation, and distance to the provincial and district centers) using spatial, simple, and partial correlation analyses. The statistical analysis revealed a negative and significant relationship between population decrease and terrain. As a result, without taking the other reasons for migration into account, the tendency of the rural migration was determined to increase as the slope, elevation, and distance to the province centers increased. The basis for a predictive model of forest villagers’ migration might thus be created by this study. Also, the driving force of migration might be revealed through quantitative modeling, and this might help create more rational development plans and programs.

## Introduction

People tend to migrate to places where the living conditions are better. Undoubtedly, it is difficult for people to decide to leave the regions they consider home and move to where they must adapt and begin new lives in a new place that may involve adjusting to cultural and lifestyle differences.

According to the International Organization for Migration (IOM), the factors forcing people to migrate can be categorized as “*economic factors*,” “*administrative factors*,” “*public services*,” “*demographic imbalances*,” “*conflicts*,” “*environmental factors*,” and “*the presence of family members who had migrated earlier*” (IOM 2013). Beginning in the 1950s, in Turkey, as was the case worldwide (Massey and Espinosa 1997; Kok and Collinson 2006; Schmidt and Sagynbekova 2008; Carr 2009; Seto 2011), people began to move from rural areas to urban centers—and even to foreign countries—in response to rural unemployment, poverty, and regional inequalities (Yalçın 2004; Filiztekin and Gökhan 2008; Özözen Kahraman et al. 2011; Sirkeci et al. 2012; Korfali et al. 2014; Akın and Dökmeci 2015; Tolay 2015).

Moreover, there have been mass waves of refugees in recent years from war-torn and terror-torn regions, such as the Middle East, to European countries. Turkey is deeply affected by these population shifts (Ignatieff et al. 2016; Gabiam 2016) and is thus well acquainted with the phenomenon of migration. Lee (1966) referred to the negative aspects of the region from which emigration occurs as the “*driving forces*.” Poverty is one of the most important such forces in the world (Sanderson 2009; Greiner 2011; Hear et al. 2012; Awumbila et al. 2014). Unsurprisingly, unemployment in rural areas—along factors behind poverty—is also a driving force behind immigration (Martin and Taylor 2003; Filiztekin and Gökhan 2008; Ranathunga 2011). When viewed from this perspective, unemployment is a factor that triggers migration, and particularly in developing countries (ILO 2010). Turkey is a developing country (WTO 2016) that faces economic crises from time to time (Cinar and Köse 2015). Unemployment resulting from economic instabilities is also a major problem for Turkey (Filiztekin and Gökhan 2008; Özözen Kahraman et al. 2011; Akın and Dökmeci 2015; Tansel et al. 2016).

In particular, unemployment, poverty, and migration in Turkey constantly impact various segments of society in differential ways, such as making a living in suburban areas, living in rural areas, and working in low-paying agriculture and forestry operations. This study examined “*forest villagers*,” the communities of people living in and adjacent to forests in Turkey. Today, approximately seven million forest villagers live in Turkey, corresponding to approximately 9% of the country’s total population (Anonymous 2016a).

Forest villagers have a significant place in Turkey’s forestry and development policies. The economic and social well-being of the forest villagers is emphasized in the Turkey’s Constitution (Anonymous 1982). Additionally, the Forest Law no. 6831 grants privileges to forest villagers in terms of employment and supplying-purchasing priority with regards to forestry activities and forest products (Anonymous 1956). Policies addressing the development of the forest villagers are also being created in the development plans, which have regularly been put into practice since 1963 (Erdönmez et al. 2010). ORKOY (forest village) projects have been conducted by the state since 1970 (Toros et al. 1997) in various regions of Turkey (Türker et al. 2003; Çoskun et al. 2007; Önal and Bekiroğlu 2011; Kızılaslan 2014). Furthermore, the sample of Mesudiye from among village-city projects (Çolakoğlu 2007; Erdönmez 2005; Erdönmez and Özden 2009) can be given as an example of activities in support of and behind the development of forest villagers. Despite all efforts, forest villagers have still been reported in the literature as the poorest rural segment of the Turkish population (Duruöz et al. 1976; Geray 1989; Atmiş et al. 2009; Erdönmez et al. 2010; Alkan and Kiliç 2014). However, forest villagers are considered part of the forest ecosystems and the most important “*labor source*” for the state forest enterprises, which own and manage almost all of Turkey’s forests on behalf of the Turkish Forest Service (approximately 28.6% of Turkey (Anonymous 2016b)). In this context, the shrinking population of youths remaining in the forest villages threatens the state’s ability to find a capable work force for forestry operations (Ok 2008; Anonymous 2012b). For these reasons, forest villagers are considered an important part of the overall population in Turkey in terms of maintaining the forestry sector and rural development.

There are no official figures on migration from the forest villages to urban areas in Turkey. Additionally, only a limited number of studies have investigated the migration of forest villagers (Özden and Birben 2007; Alkan 2014; Özden and Erkan Buğday 2015). Topography is considered the main driving force. Although rarely indicated in the literature, topography is an important factor contributing to the decision to move. Topography is also an important factor that directly affects the distribution of forest, the environment, and human interactions (Cohen and Small 1998; Small and Cohen 2004; Wu and Yao 2010; Bajat et al. 2011; Milan and Ho 2014; Telbisz et al. 2014; Telbisz et al. 2015; Zhang et al. 2015; Kummu et al. 2016; Telbisz et al. 2016). Therefore, it is considered to be the main factor behind long-term migration patterns and to nurture the potential for future migration.

The objectives of this study were to investigate the reasons related to the topographical factors behind the immigration of people from rural areas and to examine the impact generated by such movement in Turkey (the case study of Kastamonu province). Additionally, the impact of terrain conditions on the emigration movement was examined through the technical and demographic data via spatial analysis. The results of the study were also expected to yield helpful feedback for developing more efficient and realistic policies for the development of rural areas and their people by means of analyzing those factors that trigger emigration.

## Materials and methods

**Fig. 1:**
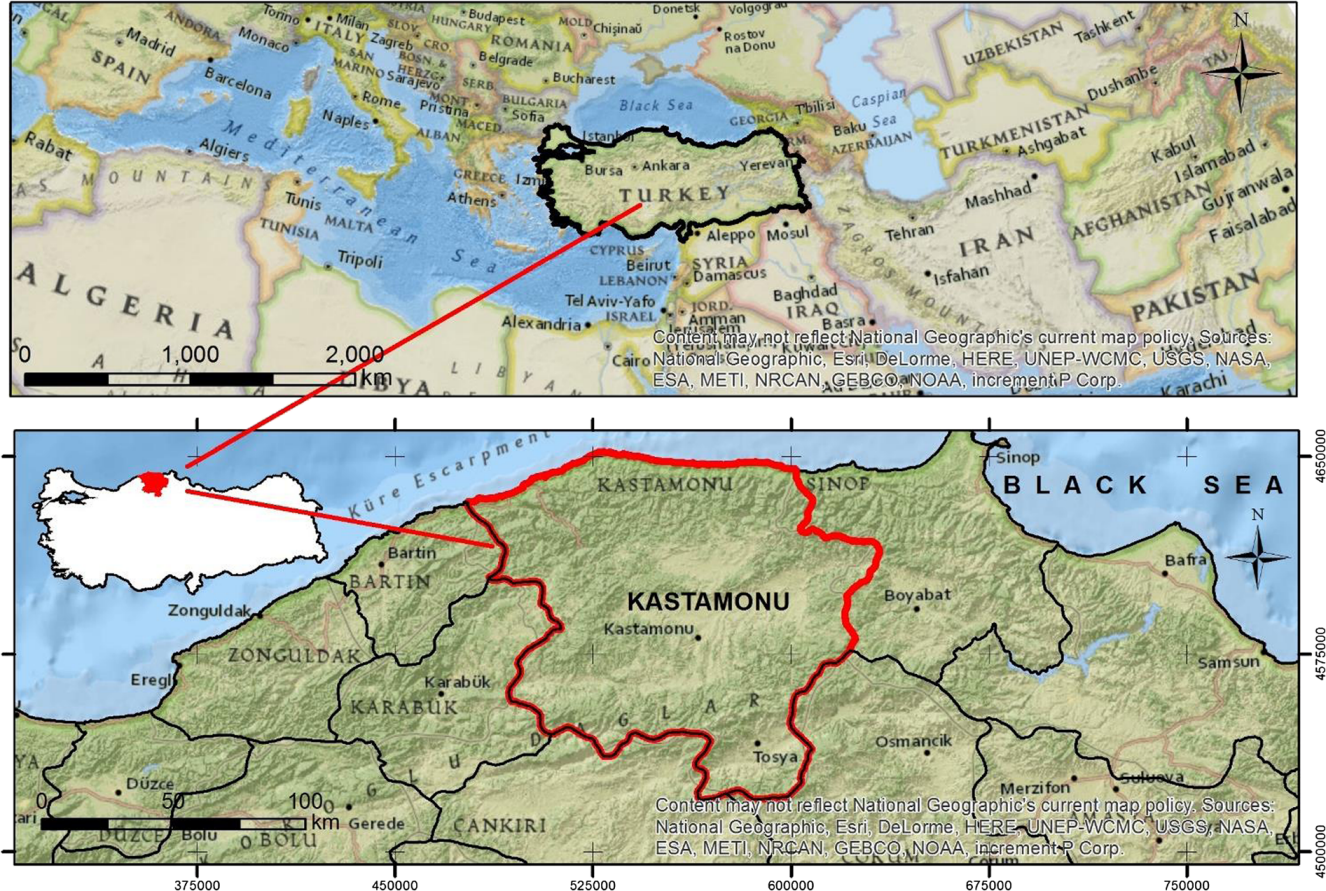
Location of study area

Forest villagers living in Kastamonu province were evaluated as the material of this study, as they make up 99% of the population living in rural Kastamonu province, and Kastamonu is the leading hub of intensive forestry activities. Therefore, Kastamonu can be viewed as an important example for forestry-related social factors in Turkey. The Kastamonu region is shown in Fig. 1. The province contains 883 villages within and 137 villages adjacent to the forests (Anonymous 2013). The demographic data of the forest villages from 1965 to 2013 were obtained from the database of the Turkish Statistics Agency (TUİK 2014). The 2013 data were indexed to the demographic data of 1965 to standardize the changes in the population of the forest villages over the 1965–2013 period. When the data were examined, the forest villages were categorized into two segments, one with increasing population and the other with decreasing population. The forest villages with increasing populations accounted for 4.5% of the total number of villages. Therefore, no re-grouping for the forest villages with increasing populations was performed. However, since forest villages with decreasing populations accounted for 95.4% of the total number of villages, those villages were examined in smaller groups. The forest villages with decreasing populations were distributed among five groups (0–20, 21–40, 41–60, 61–80, and 81–100%, with the interval showing the percentage lost), as the population decrease in the forest villages varied between 1 and 95%. By this method, the relationship between the distribution of the forest villages’ spatial locations and the distances from the provincial center and the rate of migration was shown on a spatial plane.

In this study, the topographical characteristics of the forest villages, such as slope, aspect, and distances from the provincial and district centers, were analyzed on a digital elevation model (DEM) to assess the effects of topography via ArcGIS 10 software. The results obtained from the slope analysis were divided into five groups (0–10, 11–20, 21–33, 34–50, and 51+) and classified based on the classification system of the International Union of Forest Research Organizations (IUFRO). The elevations of the village locations were analyzed via DEM, and the distribution of the forest villages based on their elevation was undertaken in 500-m intervals. The aspect analysis was performed taking into account all the cardinal points (north (N), south (S), east (E), and west (W)) and intercardinal points (northeast (NE), northwest (NW), southeast (SE), and southwest (SW)). The results of the DEM, slope, and aspect analyses were compared in numeric terms with the population changes of the forest villages over the most recent 48 years.

The slope, aspect, and elevation of the forest villages and their distance from the provincial and district centers are parameters representing their geographic positioning in general. These parameters were used to analyze the relationships between the population change ratios of the forest villages by means of Pearson and partial correlation coefficients. Before analyzing the correlation, Kuzsökü village was omitted from the data set because of extreme data. Pearson and partial correlation coefficients were used to determine the relationship among the characteristics of the forest villages and the decrease in forest village populations.

## Results

**Fig. 2:**
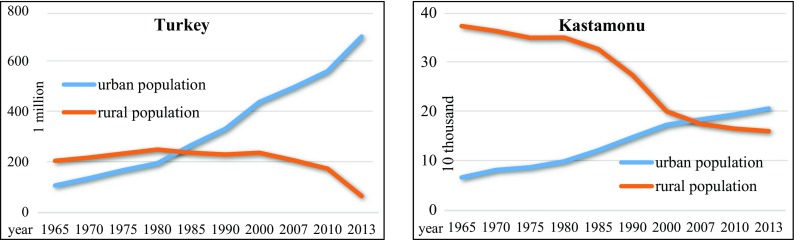
Changes in the rural and urban populations in Turkey and Kastamonu province

Changes in the rural and urban populations in both Turkey and Kastamonu over time are shown in Fig. 2.

According to the official statistics for the year 2013 (TUİK 2014), 91.35% of the overall Turkish population lived in urban areas and 8.65% lived in rural areas, whereas 56.25% of the Kastamonu population lived in urban areas and 43.75% lived in rural areas. An analysis of the temporal change in Turkey’s rural population showed that this population decreased from 65.57 to 8.65% during the 1965–2013 period. The decrease in the rural population was remarkable. As Fig. 2 shows, in 2013, there appeared to be a sudden decrease from 22.72 to 8.65% in the rural population. The new legislation on municipalities (Anonymous 2012a) can be expressed as the reason for this dramatic decrease. As a result of the new legislation concerning municipalities in Turkey, 14 provinces were granted “*metropolitan*” status, resulting in the legal statuses of all their villages also being upgraded to “*neighborhoods*.” However, the people who live in the forest villages who became neighborhoods in these metropolitan areas are still considered forest villagers under this law. Therefore, the rural population, which actually constituted approximately 20% of Turkey’s population, has changed to 8.65% in the official statistics since 2013. However, since Kastamonu province was not included in this new legislation, this dramatic decrease was not observed in its rural population.

When the rural-urban population shift in Turkey and Kastamonu was separately examined, emigration from rural areas to urban centers was seen as the root cause of the urban and rural populations arriving at an equilibrium in Turkey over the 1980–1985 period and in Kastamonu in the mid-2000s. Moreover, it has been concluded that the difference between the urban and rural populations in Kastamonu was less than the difference in Turkey overall in the 2010s.

**Fig. 3:**
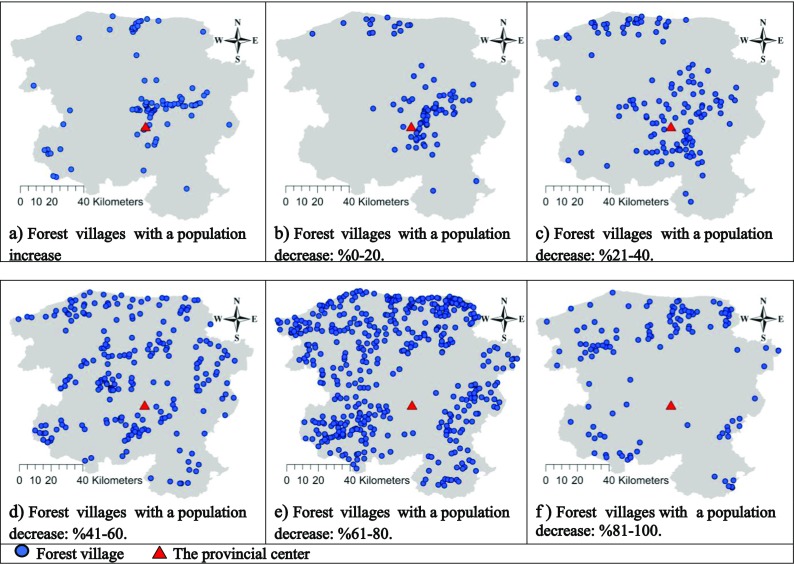
The changes in forest villages’ populations on the spatial plane

The forest villages’ increasing populations and differential rates of population decreases are shown separately on the map of Kastamonu below to highlight the changes in the forest villages’ populations on the spatial plane (Fig. 3).

When the changes in the forest villages’ populations between 1965 and 2013 were examined on maps, those villages with increased populations were typically located around the center of the province. In addition, the forest villages with the highest migration rate (81–100%) were the farthest from the province center and distributed around the province’s administrative border. Overall, the rate of migration increased with distance from the center of the province, except for those forest villages with a population decrease of 41–60%. The forest villages belonging to this group were scattered all around the province.

After the study of the spatial distribution of forest villages, the slopes of the forest villages’ spatial locations were analyzed, and five groups were created. The distribution of the forest villages to the slope groups is presented in Table 1.

**Table 1:** The distribution of the population changes of the forest villages in Kastamonu province based on slope groups

	0–10% slope	11–20% slope	21–33% slope	34–50% slope	51+% slope	Total
Population increase	2	11	5	8	20	46
0–20% decrease	6	14	20	17	18	75
21–40% decrease	7	22	23	29	49	130
41–60% decrease	13	30	41	59	70	213
61–80% decrease	34	65	78	107	156	440
81–100% decrease	6	26	19	35	30	116
Total	68	168	186	255	343	1020

According to the results of the slope analysis, 7% of the forest villages were located in the 0–10% slope group, 16% in the 11–20% slope group, 18% in the 21–33% slope group, 25% in the 34–50% slope group, and 34% in the 51+% slope group. The results of this analysis indicate that 59% of the forest villages belonged to steep (34–50% slope) and very steep (51+% slope) slope groups. A greater number of forest villages were located on relatively inclined slopes than those on flat or slightly inclined slopes.

The forest villages’ spatial locations were also analyzed using eight aspect groups. The distribution of the forest villages among the aspect groups is presented in Table 2.

**Table 2:** The distribution of the population changes of the forest villages in Kastamonu province using aspect groups

	N	NW	E	SE	S	SW	W	NW	Total
Population increase	8	4	8	6	6	4	6	4	46
0–20% decrease	11	11	9	12	9	4	9	10	75
21–40% decrease	20	17	17	24	14	6	11	21	130
41–60% decrease	27	17	28	33	33	27	22	26	213
61–80% decrease	57	52	46	74	78	53	35	45	440
81–100% decrease	15	10	14	14	21	16	12	14	116
Total	138	111	122	163	161	110	95	120	1020

Since people seek to use daylight as much as possible, those areas with southward aspects were generally preferred for settlement purposes. Because the settlement areas with northward aspects could use daylight for a shorter part of the day and experienced more frost in the winter, it was assumed that such locations had a negative impact on daily living and increased the tendency to migrate; consequently, an aspect analysis was performed on the forest villages. Table 2 indicates that 42.54 and 36.17% of the forest villages located in Kastamonu province had southward and northward aspects, respectively.

The results of the analysis show that 13.53% of the forest villages settled in areas oriented northward, 10.88% in northeastern-oriented areas, 11.96% in areas oriented eastward, 15.98% in southeastern-oriented areas, 15.78% in southern-oriented areas, 10.78% in southwestern-oriented areas, 9.31% in westward-oriented areas, and 11.76% in northwestern-oriented areas.

When the groups of villages and aspects were examined, the largest number of the forest villages were found in the southward and southeastern-oriented areas and in the 61–80% decrease group. The analysis results show that the test-oriented areas were found to be the least preferred for settling.

The elevations of the forest villages’ spatial locations were analyzed, and four groups were created. The distribution of the forest villages among the elevation groups is presented in Table 3.

**Table 3:** The distribution of the population changes of the forest villages in Kastamonu province by elevation group

	0–500 masl	501–1000 masl	1001–1500 masl	1501–2000 masl	Total
Population increase	6	32	8	−	46
0–20% decrease	10	55	10	−	75
21–40% decrease	21	55	53	1	130
41–60% decrease	37	91	85	−	213
61–80% decrease	111	161	164	4	440
81–100% decrease	17	53	39	7	116
Total	202	447	359	12	1020

According to the results of the DEM analysis, 19.80% of the villages were situated at an altitude of 0–500 masl, 43.82% at approximately 501–1000 masl, 35.20% at approximately 1001–1500 masl, and 1.18% at approximately 1501–2000 masl.

When the groups of the forest villages and the elevation groups were compared, the largest number of forest villages were found in the elevation groups of 501–1000 and 1001–1500 masl and in the 61–80% decrease group. Based on the analysis result, the elevation group of 1501–2000 masl was found to be the least preferred for settling.

A Pearson correlation analysis was performed to determine the relationship between the decreases in the forest villages’ populations and the topographical characteristics of the forest villages, such as slope, aspect, elevation, and distances from province and district centers. The results of the Pearson correlation analysis are presented in Table 4.

**Table 4:** Correlations between the changes in population and slope, aspect, elevation, and distance

		Population (Pp)	Slope	Aspect	Elevation	District (D)	Province (P)
			(S)	(A)	(E)		
Population	Pearson correlation	1	−0.155^**^	−0.029	−0.070^*^	−0.134^**^	−0.357^**^
	Sig. (2-tailed)		0	0.356	0.025	0	0
	Number	1019	1019	1019	1019	1019	1019

**Correlation is significant at the 0.01 level (2-tailed)

*Correlation is significant at the 0.05 level (2-tailed)

From the results of the Pearson correlation analysis, no significant relationship was identified between decreases in the forest villages’ populations (1965–2013) and the aspects of the forest villages. However, a significant relationship (95% confidence interval) was also found among the decreases in forest village populations and the slopes of particular village locations, the elevations of the village locations, and the distances to the district and provincial centers. The decreases in forest village populations had the strongest correlation with distance to the provincial center. Statistically, the correlation was medium in strength and negative (−0.357). It might be said that as the distance from the provincial center increased, the population of the forest villages decreased. Whereas the correlation between the decreases in forest village populations and the distances to the provincial center was also found to be medium in strength and negative, the correlation between the decreases in forest village populations and the distances to the district center was determined to be low in strength and negative (−0.134). There was a low and negative correlation between decreases in forest village population and both the elevation (−0.070) and the slope of the villages’ locations (−0.155).

The characteristics of the forest villages (slope, aspect, elevation, and distances from the centers of the province and district) were the result of topographical characteristics, and each characteristic (parameter) could be assumed to affect one another at the same time. As a result, a partial correlation analysis was performed between the decreases in forest village populations and one single such characteristic, while controlling for the other characteristics. The results of the partial correlation analysis are presented in Table 5.

**Table 5:** Correlations between the change in population and slope, aspect, elevation, and distance

Control variables		Correlation	Significance (2-tailed)	df
Aspect and elevation	Population–province	−0.475^*^	0	1015
Aspect and elevation and slope	Population–province	−0.450^*^	0	1014
Slope and province and district and aspect	Population–elevation	−0.349^*^	0	1013
Aspect and elevation	Population–slope	−0.174^*^	0	1015
Elevation and province and district and slope	Population–aspect	−0.012	0.714	1013
Elevation and province and aspect and slope	Population–district	0.049	0.121	1014

*Correlation is significant at the 0.05 level (2-tailed)

A significant relationship between the decreases in forest village populations and distance to the provincial center (−0.475) was identified, while controlling for the aspects and elevations of the villages’ locations using a partial correlation analysis. Additionally, when the slopes of the villages’ locations were added to the control group, another significant relationship between the decreases in the forest villages’ populations and the distances from the provincial center was found. Statistically, the correlation was medium in strength and negative (−0.450). Although in the Pearson correlation analysis, a correlation between the decreases in forest village populations and the elevation of the villages’ locations was determined to be low in strength and negative (−0.134), in the partial correlation analysis, a slightly stronger correlation was found (−0.349), when controlling for the aspects and slopes of the villages’ locations and the distances from the provincial and district centers. A correlation between the decreases in the forest villages’ populations and the slopes of the villages’ locations was determined to be low in strength and negative (−0.174), while controlling for the aspects and elevations of the villages’ locations, as in the results of the Pearson correlation analysis. Although a simple correlation was found between the population and distance to the district centers, no correlation was found in a partial correlation analysis when controlling for the elevations, aspects and slopes of the villages’ locations and distances from the provincial centers. No correlation was found between decreases in forest village populations and the aspects of the villages’ locations in both the partial and Pearson correlation analyses.

## Discussion and conclusion

Despite the difficulties faced by people living in the rural regions during the migration process and its aftermath, their migration can be understood as a reaction to the economic, social, political, ecological, and topographical diversity they faced. In this study, ignoring the other factors, the migration phenomenon experienced during the past 48 years was analyzed in relation to the topographical factors, and as a result of the statistical evaluation, topography was determined to be a definitive factor in the population (Cohen and Small 1998; Telbisz et al. 2014; Milan and Ho 2014; Telbisz et al. 2015; Telbisz et al. 2016; Zhang et al. 2015).

Kastamonu province lost approximately half of its rural population between the years 1965 and 2013. An examination of the change in population of the forest villages in this period was examined; only 4.5% of the forest villages were determined to have had a population increase, whereas 95.5% were found to have had their populations fall. Additionally, 43.13% of the forest villages lost population in the range of 61–80%. In this study, the slopes, elevations, aspects, and distances from the provincial and district centers of the forest village locations were determined to be adverse topographical parameters that led to migration.

As a result of the spatial analysis, among the forest villages, the most affected by migration were those located farthest from the provincial center and also those located close to the borders of neighboring provinces. This finding is remarkable. Thus, it could be said that there was a relation between the distance from the provincial center and the migration rate (Wu and Yao 2010). However, those forest villages with increasing populations were concentrated around the provincial center. These results demonstrated that the forest villages exploited opportunities arising in and around the provincial center, such as education, health, transportation, infrastructure, and employment opportunities. Therefore, it might be stated that people in forest villages close to the provincial center tended to migrate less than those in forest villages far from the provincial center.

This study found that 33.62% of the forest villages were located on very steep terrain. When the aspects of the forest villages’ locations were examined, it was determined that 31.76% were settled on southern-facing and southwestern-facing aspects. In contrast to the distribution of settlements around the world (Cohen and Small 1998) but similar to the results of the local study of Telbisz et al. (2014), the vast majority of forest villages (79%) were found to be situated between 501 and 1500 masl in this local study.

A simple correlation was performed between population changes in forest villages and the factors’ slope, aspect, elevation, and distance from the provincial and district centers, and the strongest correlation was found between the population changes in the forest villages and the distances from the provincial center (Bajat et al. 2011). The correlation between the population changes in the forest villages and the distances from the provincial center supported the results of the spatial analysis. A slightly weaker correlation was found between the population and the variables of slope, elevation, and distance from the district centers. However, no significant relationship was found between the population and the direction of the forest village.

When the partial correlation between the population changes in the forest villages and each of the topographical parameters was sought, stronger correlations were found. Therefore, the slope, elevation, and distances from the provincial and district centers could be hypothesized to weaken each other’s relationships with the population changes. In this study, the slopes and elevations of the villages’ locations and the distances from the provincial and district centers were shown to affect the migration of forest villagers living in Kastamonu province.

At the same time, this study showed that the topographic parameters should be examined in detail during the evaluation of the factors causing the migration as well as demographic events. As being the case in this study, the official databases kept up to date by the states are important tools for the studies focusing on the social events. It is necessary to keep the databases’ data entry and upkeep procedures stable in order to ensure continuity of long-term studies and monitoring of social, economical, and ecological events. Additionally, this study was conducted in an important area in terms of forestry and forest villagers. The provinces with a large number of forest villages should be primarily selected in the projects regarding development and support of the rural population. This suggestion should be heeded in national development plans.

The state forest enterprises, with an organizational structure covering all of Turkey, should take the topographic characteristics of forest villages into account in forest management activities. As in the case of Kastamonu province, the people living in the forest villages situated at high elevations, on steep slopes, and far from the provincial center(s) should be employed for the duration of 12 months each year. Forest villagers should be encouraged in the production of non-wood forest products as well as to tend livestock to generate additional income and lessen the negative effects of the topography, thereby strengthening their economic stability. However, ecotourism activities can be undertaken by evaluating the scenic values of the high elevations and pastures. By this means, the negative effects of the topography can be valued as attractive forces in ecotourism and similar activities.

In this study, migration was examined in terms of topographical features, such as slope, aspect, and distance from the provincial and district centers. In further studies, the relationship between the different parameters of the topography (e.g., land use) and various characteristics of the population (e.g., age, income level, cultural structure) could be examined. Population prediction models could be created in relation to many different parameters using geographical information systems methods at both the local and global levels.
